# Integrated pharmacology and validation reveal Formononetin upregulates GSTM2 to modulate prostate cancer immune microenvironment via ROS/STAT3-driven chemokine reprogramming

**DOI:** 10.3389/fimmu.2026.1964912

**Published:** 2026-10-23

**Authors:** Tianlun Yu, Yu Zhou, Fangkai He, Yufan Wu, Shiyun Tang, Zhouhan Zhu, Wei Qian

**Affiliations:** 1 Department of Clinical Laboratory, Kunshan Rehabilitation Hospital, Kunshan, China; 2 Central Laboratory, Kunshan Hospital of Traditional Chinese Medicine, Kunshan Affiliated Hospital of Nanjing University of Chinese Medicine, Kunshan, China; 3 Department of Urology, Kunshan Hospital of Traditional Chinese Medicine, Kunshan Affiliated Hospital of Nanjing University of Chinese Medicine, Kunshan, China; 4 Kunshan Key Laboratory of Immune Regulation in Health and Disease, Kunshan, China

**Keywords:** prostate cancer, formononetin, GSTM2, network pharmacology, biochemical recurrence, oxidative stress, chemokine

## Abstract

**Background:**

Prostate cancer (PCa) is responsible for a substantial proportion of male cancer cases worldwide. Biochemical recurrence (BCR) representing a critical challenge in its disease management. Formononetin, a natural isoflavone derived from traditional Chinese medicine, has demonstrated potential anticancer properties. We sought to determine the translational potential of Formononetin-targeted molecules in PCa.

**Methods:**

A list of putative targets of Formononetin were identified through five public databases. Overlapping candidates underwent further refinement using an ensemble of bioinformatics tools, encompassing differential expression profiling, co-expression network construction, and multiple machine-learning classifiers. Docked complexes and complementary analyses were subjected to evaluate the binding stability. To functionally validate GSTM2, we assessed its functional role on PCa cell growth, motility, and invasiveness *in vitro*, as well as the expression of CXCL10 and CCL22 chemokines.

**Results:**

Integrative analysis identified 413 Formononetin targets, with overlapped 335 PCa-associated genes. Through weighted gene co-expression network analysis and differential expression analysis, 22 candidate genes were identified as Formononetin-related genes in PCa. Machine learning algorithms and survival analysis further suggested GSTM2 as the most important target. GSTM2 was positively correlated with enhanced immune infiltration and sensitivity to Bicalutamide. Single-cell analysis revealed that GSTM2-positive malignant cells exhibited upregulated glutathione metabolism and suppressed respiratory burst pathways. Stable complex formation between Formononetin and GSTM2 was demonstrated by computational docking. Cell-based assays demonstrated that Formononetin upregulated GSTM2 and inhibited PCa malignancy in a GSTM2-required manner. In terms of mechanism, Formononetin attenuated ROS levels and suppressed STAT3 phosphorylation via GSTM2, leading to reprogramming of chemokine expression characterized by upregulated CXCL10 and downregulated CCL22.

**Conclusion:**

Formononetin reprograms chemokine expression through the GSTM2-ROS-STAT3 axis to regulate the immune microenvironment, suggesting that it might have immune-modulating effects and is worth further *in vivo* studies.

## 1 Introduction

Biochemical recurrence (BCR) can happen in up to 46% of prostate cancer (PCa) patients within ten years after radical treatment, but right now, there are no approved drugs specifically for this post-surgery stage (1, 2). Androgen deprivation therapy is currently the standard approach for high-risk cases. It can buy some time, but the cost is metabolic toxicity and inevitable development of castration-resistant prostate cancer (CRPC) (3). Is there a well-tolerated natural compound that could fill this gap? Traditional Chinese Medicine (TCM) active ingredients have exhibited unique properties in prevention and treatment, due to their multi-targeting, low toxicity, and synergistic properties (4–6). Here, we focus on Formononetin, which is an isoflavone from *Astragalus*. It is known for its antioxidant activity (7–9), its specific molecular targets and regulatory networks in PCa were lack of systematic exploration.

Network pharmacology could help decipher the systematic elucidation of the multi-target synergistic mechanisms of TCM active ingredients. In cancer research, bioinformatics algorithms, such as weighted gene co-expression network analysis (WGCNA), machine learning models are broadly adopted. By integrating these technical approaches, we can screen for potential biomarkers and actionable drug targets. While computational docking and dynamics were employed to characterize the binding stability of small molecules to target proteins at the atomic level. Under this approach, we identified a key target gene for Formononetin in PCa. As a mu class glutathione S-transferase, GSTM2 encoded enzyme catalyzes glutathione conjugation to electrophilic substrates, thereby participating in the detoxification of carcinogens, environmental toxins, and oxidative stress products, while maintaining intracellular redox balance (10–12). In a whole-genome methylation analysis of 58 prostate tumor tissues, Angulo et al. found *GSTM2* promoter hypermethylation in 79.3% of cases, which independently predicted BCR after radical prostatectomy (13). Moreover, GSTM2 is significantly overexpressed in enzalutamide-resistant CRPC and drives resistance to second-generation androgen receptor inhibitors by counteracting oxidative stress and activating the p38 MAPK pathway, suggesting its roles may vary across disease stages (14). As a key antioxidant enzyme, GSTM2 regulates intracellular ROS scavenging (15). ROS, acting as second messengers, activate JAK/STAT signaling, influencing tumor cell proliferation, survival, and immune cell recruitment (16, 17). The STAT1 and STAT3 are the most frequently activated by ROS among STAT family members. They may alter the immune microenvironment in a way of regulating downstream chemokines. They led in distinct directions: STAT1 mainly upregulates CXCL10 to recruit CD8^+^ T, NK and Th1 cells (18–20), whereas STAT3 upregulates CCL22 to recruit Treg and Th2 cells (21, 22). To some extent, the CXCL10/CCL22 ratio reflects the differences of immune cell infiltration (23). However, whether GSTM2 influences early-stage PCa progression through the ROS/STAT/chemokine axis remains unexplored, and no study has investigated GSTM2 as a direct target of Formononetin.

## 2 Methods

### 2.1 Targets of formononetin

A heterogeneous data acquisition strategy was employed to identify putative targets of Formononetin. Initially, TCMSP (https://www.tcmsp-e.com/) was queried for the keyword “Formononetin” (24), yielding 39 related targets, which were subsequently manually curated to convert them into their corresponding human gene symbols. Concurrently, targets were acquired from CTD (https://ctdbase.org/) and HERB (http://herb.ac.cn/) databases (25, 26), resulting in 91 and 34 targets, respectively. To further expand the target landscape, structure-based computational predictions were conducted via SwissTarget (https://swisstargetprediction.ch/) and PharmMapper (https://www.lilab-ecust.cn/pharmmapper/index.html) web servers (27, 28). Briefly, the canonical SMILES string and the SDF V2000 structural file (both obtained from PubChem, CID: 5280378) were uploaded to these platforms, which predicted 101 and 239 potential targets, respectively.

### 2.2 Access and analysis of online cancer database resources

Using “prostate cancer” as the search term, PCa-related genes were obtained from GeneCards (https://www.genecards.org/) and OMIM (https://www.omim.org/) databases. A relevance threshold of 1.0 was applied in the GeneCards. This approach yielded 7,508 and 180 genes from each database, respectively, which after deduplication resulted in a final set of 7,515 unique genes. Transcriptome data were sourced from multiple sources, including DKFZ_2018, TCGA_PRAD and GSE54460 (Information in **Supplementary Table S1**). For different expression genes (DEGs) analysis, the result of TCGA_PRAD cohort was using “DESeq2” package based on raw count data. For WGCNA, gene expression in TPM format from the DKFZ_2018 cohort was subjected to filtering, retaining only genes with an inter-sample standard deviation > 0.5 to exclude those with low variation. An approach involving β values from 1 to 30 was employed, with the final parameter selected by balancing scale-free R² and mean connectivity to meet the network assumption. The optimal power (12) was selected as the value that maximized R² while maintaining sufficient connectivity, ensuring both network fitness and biological interpretability. Subsequently, modules with a correlation coefficient |r| > 0.3 with BCR were extracted.

### 2.3 Enrichment characterization and PPI network analysis of candidate targets

The R package “clusterProfiler” was applied for functional annotation of the candidate genes, with GO annotations from “GO.db” and KEGG pathways retrieved via “createKEGGdb” (29). Pathway visualizations were generated using “pathview” (30). For PPI analysis, candidates were submitted to STRING (https://string-db.org) database, and interactions passing the confidence threshold of 0.4 were retained (31). The node connectivity (degree) was recalculated to construct the final PPI network.

### 2.4 Machine learning algorithms

LASSO Cox regression, random survival forest, and SVM-RFE were employed as three complementary machine learning approaches. (i) LASSO Cox regression was performed to select the most robust prognostic genes from the candidate set by “glmnet” R package (32–34). LASSO regression applies a penalty to shrink coefficients, discarding variables with zero coefficients while retaining the rest. The optimal penalization parameter (λ) was selected via 10-fold cross-validation using the λ.min criterion. (ii) “randomForestSRC” R package was used for random survival forest model. Bootstrap resampling was applied to generate training subsets, with model performance internally validated against out-of-bag (OOB) samples. The Nelson-Aalen estimator was used to estimate cumulative hazard, and the final prediction was aggregated from all trees via weighted voting. The predictive performance of the model was reflected by the C-index (35). (iii) SVM-RFE, an embedded machine learning algorithm for binary classification, was applied to screen key features using the mSVM-RFE function. The algorithm recursively eliminates features with the smallest contribution to the classification hyperplane, and the optimal subset was determined via cross-validation (36). All candidate genes were included as initial features with default settings.

### 2.5 Prognostic validation of hub genes

The hazard contribution of each hub gene’s expression to BCR-free survival was estimated by univariate Cox regression, forest plots were generated to present the results. Patients were dichotomized by median expression, and log-rank comparison of survival curves was performed in Kaplan–Meier analysis.

### 2.6 Immune infiltration and drug sensitivity analysis

The tumor microenvironment (TME) characteristics were generated via the “estimate” R package (37). The ssGSEA method was also served to quantify the composition of diverse immune cell types. We adopted “oncoPredict” R package to assess chemo-response to Bicalutamide (38). The GDSC2 database version 8.5 was employed as a training dataset. Genes with low variation across samples, defined as those with a coefficient of variation below 0.2, were filtered out to improve the robustness of the analysis.

### 2.7 Single-cell transcriptomic data

Single-cell RNAseq data from PCa samples were obtained from GSE157703 (39). Raw count matrices were processed with Seurat V5, including quality filtering (cells with <200 genes or >25% mitochondrial reads), variable feature selection (2,000 genes via vst) following log-normalization (scale factor = 10,000), scaling, and Harmony batch correction. Dimensionality reduction was performed using PCA and UMAP on was constructed to cluster cells at a resolution of 0.5. The Human Primary Cell Atlas served as a guide for SingleR-based cell-annotation, followed by manual refinement based on canonical marker genes (40, 41). For functional annotation, we ranked all expressed genes using FindMarkers function with Wilcoxon rank-sum test (*p* < 0.05), and the GSEA was conducted using “clusterProfiler” based on those genes between GSTM2-positive and GSTM2-negative subsets.

### 2.8 Structural modeling

A 2D structure of Formononetin was obtained from PubChem, minimized using Chem3D 23.1.1, and exported to mol2 format for subsequent docking. The PDB (PDB ID: 3GUR) provided the crystal structure of ligand free GSTM2, cleaned with PyMOL 2.6 (water/phosphate removed). AutoDockTools performed hydrogenation, charge assignment, and rotatable bond definition (42); We performed docking using AutoDock Vina 1.2.5 with a grid box covering the active site and selected the lowest-energy pose (43).

The Amber99SB-ILDN (protein) and GAFF2 (ligand) force field was employed for molecular dynamics (MD) simulations, and the RESP charges were generated using sobtop_1.0. The complex was placed in a cubic box (1.0 nm) filled with TIP3P water and neutralized by adding 0.15 M NaCl. PME was used for long-range electrostatics (cutoff: 1.2 nm), bonds constrained with LINCS, and step size set to 2 fs (44, 45). After minimization, the system was equilibrated (1 ns NVT, 1 ns NPT) at 300 K/1 bar with restraints, then run for 100 ns production. Post-simulation analyses (RMSD, RMSF, Rg, SASA, and FEL) were performed using GROMACS built-in tools.

### 2.9 Cell culture and reagents

Human PCa cell lines LNCaP and VCaP, obtained from Boster Company (CX0095 and CX0246), were maintained in their respective media (RPMI-1640 and DMEM) with 10% FBS, under standard conditions. Formononetin (HY-N0183, MCE), GSTM2 polyclonal antibody (55474-1-AP, Proteintech), STAT3 polyclonal antibody (10253-2-AP, Proteintech), Phospho-STAT3 (Tyr705) antibody (9131, Cell Signaling Technology), STAT1 polyclonal antibody (10144-2-AP, Proteintech), Phospho-STAT1 (Tyr701) monoclonal antibody (33-3400, Invitrogen), β-actin antibody (sc-47778, Santa Cruz), CXCL10 ELISA Kit (EK168, MultiSciences), and CCL22 ELISA Kit (E-EL-H0029, Elabscience) were obtained from the indicated vendors. Two siRNA duplexes targeting human *GSTM2* were synthesized by GenePharma (Shanghai, China), and their information is summarized in **Supplementary Table S2**. CALNP™ RNAi *in vitro* (DN001, D-Nano) served as the transfection reagent.

### 2.10 CCK-8 proliferation and colony formation assay

For CCK-8 assay, 1500 cells were plated in each well. At predetermined intervals, 10 µL of CCK-8 reagent (BS350A, Biosharp) was added, followed by a 2-hour incubation, the absorbance was recorded at 450 nm. To evaluate colony-forming ability, cells were seeded at 800 cells per 35-mm dish and cultured for 10–14 days. Colonies were then fixed (4% paraformaldehyde, 15 min), stained with 0.1% crystal violet, and enumerated manually.

### 2.11 Cell migration and invasion assays

For migration, 5×10^4^ cells suspended in 200 µL serum-free medium were added to the upper compartment of uncoated Transwell inserts, while the lower chamber was filled with 650 µL of 20% FBS-containing medium. The lower surface was stained with crystal violet at 24 h post-seeding. For invasion, inserts were pre-coated with Matrigel, and cells were treated identically but incubated for 48 h.

### 2.12 Reactive oxygen species measurement

For flow cytometric detection of intracellular ROS, cells from different treatment groups were harvested and incubated with 10 μM DCFH-DA (S0033S, Beyotime) for 30 minutes under dark conditions. We then measured FITC fluorescence by flow cytometry and analyzed the data.

### 2.13 Quantitative RT-PCR and ELISA

Total RNA was extracted using the Super FastPure Cell RNA Isolation Kit (Vazyme, RC102). Reverse transcription and qPCR were performed using the Reverse Transcription Kit (Biosharp, BL699A) and Universal SYBR qPCR Master Mix (Biosharp, BL697A), respectively. The primer information is listed in **Supplementary Table S3**. For cytokine detection, conditioned media were harvested following a 24-hour treatment period, secreted CXCL10 and CCL22 levels were measured, and concentrations interpolated using standard curves according to the kit protocols.

### 2.14 Statistical analysis

For intergroup comparisons of bioinformatics analyses related to publicly available transcriptomic sequencing data, the nonparametric Wilcoxon rank-sum test was used. All *in vitro* experiments data are presented as mean ± SD. A one-way ANOVA analysis was first performed to confirm the presence of significant overall differences between groups, followed by planned comparisons with Bonferroni correction. Statistical computations were conducted in R 4.3.1.

## 3 Results

### 3.1 Identification of formononetin targets associated with PCa progression

**Figure 1A** illustrates the 3D structure of Formononetin (https://pubchem.ncbi.nlm.nih.gov/compound/5280378). The compound exhibited high oral bioavailability (69.67%) and moderate drug-likeness (DL = 0.21) according to TCMSP. After deduplicating, integrative target prediction across five databases (TCMSP, CTD, HERB, SwissTarget and PharmMapper) generated 413 unique Formononetin targets (**Figure 1B**; **Supplementary Table S4**), with three genes being concurrently predicted by all five databases, indicating their robust candidacy. Of the 413 targets, 335 overlapped with PCa-related genes (**Figure 1C**), highlighting the broad therapeutic potential of Formononetin in PCa.

**Figure 1:**
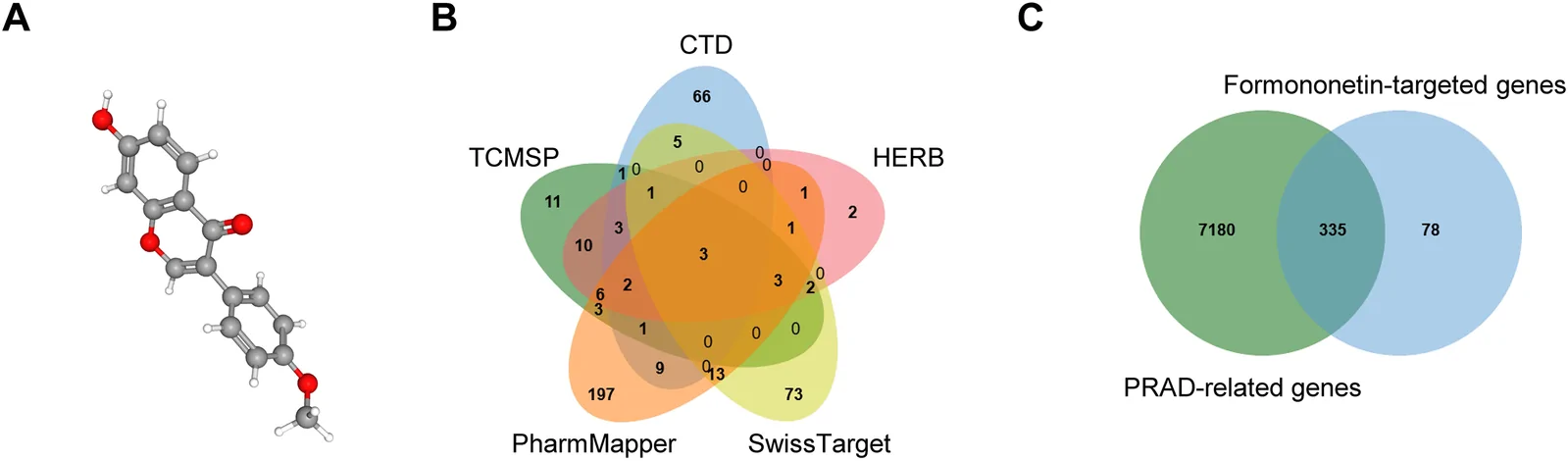
Pharmacological network. **(A)** 3D structure of Formononetin (C_16_H_12_O_4_). **(B)** Target prediction from five databases. **(C)** Overlap of Formononetin targets and PCa-related genes.

Differential expression analysis of 19,504 protein-coding genes from TCGA_PRAD (483 primary tumors vs. 51 normal tissues) identified 3,070 DEGs at |log_2_FoldChange| > 1 and adjusted *p*-value < 0.05 (**Figure 2A**; **Supplementary Table S5**). In DKFZ_2018 cohort (n = 105), WGCNA was performed on the 11,647 genes retained after filtering. Construction of a scale-free network required selection of a soft-thresholding power (**Figures 2B, C**; **Supplementary Table S6**), and the topological overlap matrix (TOM) was subsequently generated using the weighted correlation coefficients. Using the dissTOM matrix for average-linkage hierarchical clustering, we then performed initial module identification via the dynamic tree-cutting method. To avoid over-division, highly similar modules were merged based on their eigengene correlations. By this process, 11,647 genes were partitioned into 22 co-expression modules; the grey cluster, which contained genes of unknown assignment, was not carried forward (**Figures 2D, E**). With correlation coefficients between module eigengenes and BCR status > 0.3 as the cutoff, 1,324 genes were retrieved from modules (**Figure 2F**; **Supplementary Table S7**). 22 candidate genes were identified from the overlap among these genes, the 3,070 DEGs, and the 335 PCa-related Formononetin targets (**Figure 2G**).

**Figure 2:**
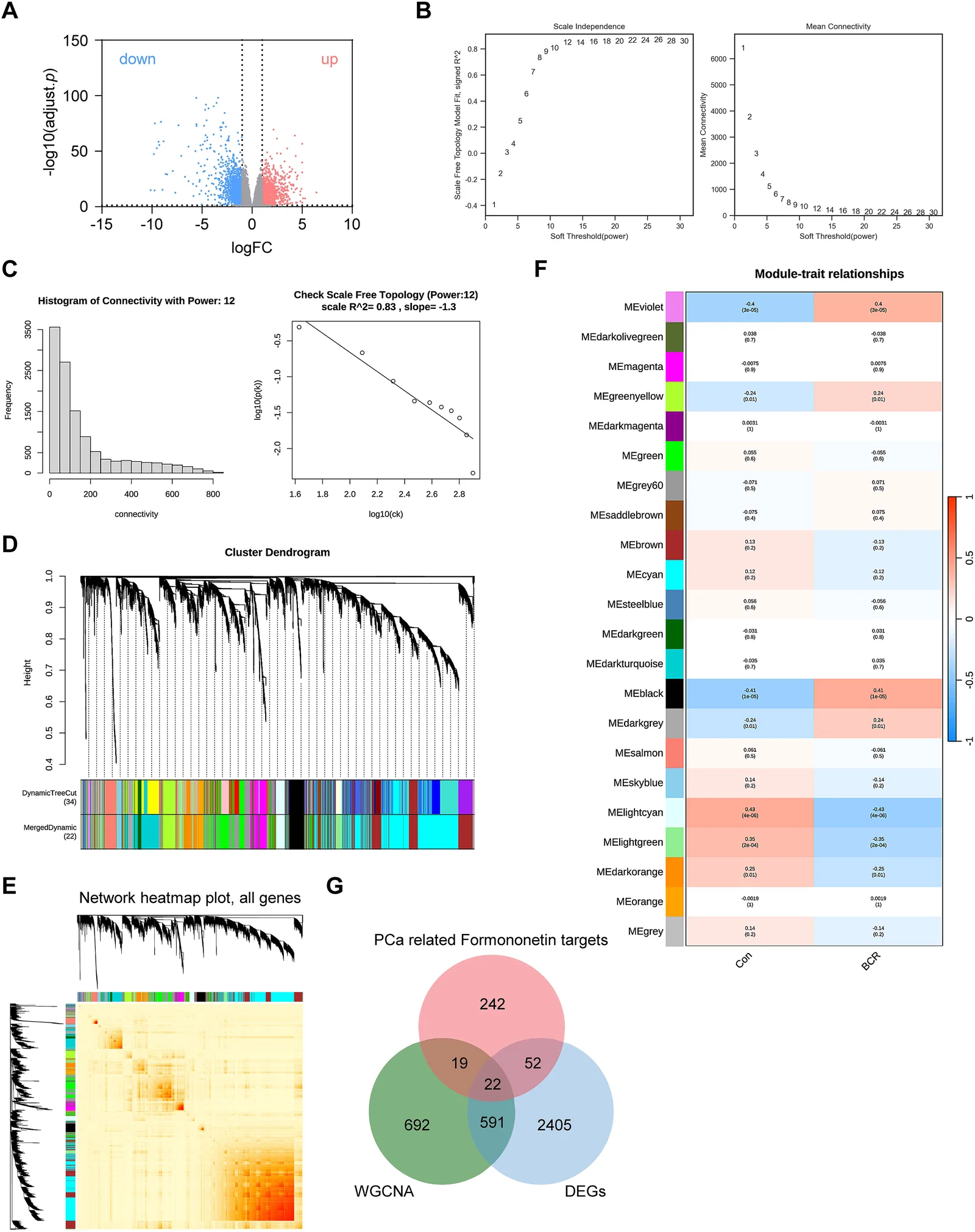
Formononetin target genes associated with PCa progression. **(A)** PCa tumor vs. normal DEGs. **(B)** Network connectivity and topology across β values. **(C)** Analysis for power selection. **(D)** Hierarchical clustering dendrogram. **(E)** TOM heatmap illustrating the network connectivity among all genes in the identified modules. **(F)** Module–trait relationships. **(G)** Overlap among PCa-related Formononetin targets, DEGs, and WGCNA module genes.

### 3.2 Functional landscape of candidate targets

As shown in **Figures 3A–C**, GO enrichment results suggested that these 22 genes were predominantly associated with xenobiotic metabolism, hormone regulation, and cell cycle progression. The top three represented biological process terms were response to xenobiotic stimulus, olefinic compound metabolic process, and flavonoid metabolic process. The main cellular component terms enriched were membrane-associated compartments, including the nuclear envelope lumen, cytoplasmic side of plasma membrane, and mitochondrial outer membrane. In terms of molecular functions, retinoid binding, isoprenoid binding, monocarboxylic acid binding, steroid binding, glucuronosyltransferase activity, and histone kinase activity were among the most significantly enriched terms. KEGG mainly mapped metabolic and cancer-related pathways (**Figure 3D**; **Supplementary Table S8**), such as Drug metabolism-cytochrome P450, Steroid hormone biosynthesis, and the cell cycle pathway. To characterize the interrelationships among the 22 candidate proteins, we assembled a PPI network. BCL2, PPARGC1A, KIT and PGR may function as hub genes for their highest connectivity (**Figure 3E**).

**Figure 3:**
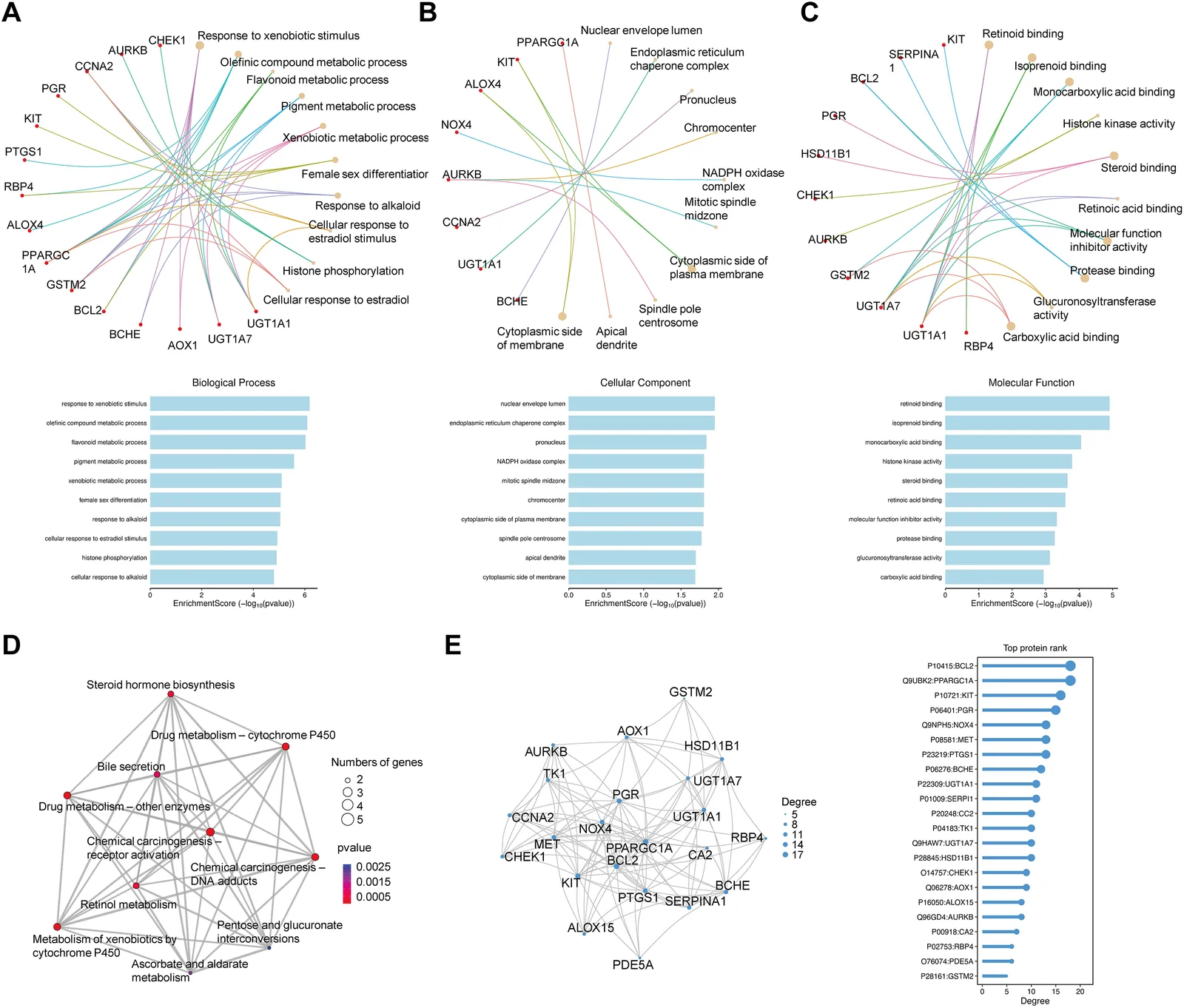
Functional and network analyses. **(A–C)** Gene–concept network visualization of GO terms. **(D)** KEGG pathway enrichment map, where each node represents a pathway and edges connecting nodes reflect the extent of shared genes between pathways. **(E)** PPI network (22 genes).

### 3.3 Screening and prognostic validation of the core target GSTM2

Complementary machine learning algorithms were further identified the most pivotal target gene from the 22 candidates. LASSO Cox regression selected 4 genes under the λ.min criterion, RSF identified 8 prognostic genes, and SVM-RFE with 10-fold cross-validation screened 9 features (**Figures 4A–F**; **Supplementary Table S9**). Overlap analysis of the three algorithms revealed two consistent genes: TK1 and GSTM2 (**Figure 4G**). Univariate Cox analysis further evaluated the predictive ability of two genes for BCR-free survival. Both TK1 and GSTM2 showed significant associations with BCR risk (TK1: HR = 2.812, 95% CI 1.787–4.423, *p* < 0.001; GSTM2: HR = 0.340, 95% CI 0.206–0.563, *p* < 0.001) (**Figure 4H**). KM-based survival analysis stratified by median expression further validated their prognostic value. In both the DKFZ_2018 and GSE54460 cohorts, GSTM2 demonstrated consistently stable prognostic performance, the high-expression group exhibited a significantly favorable BCR-free survival. In contrast, TK1 exhibited weaker and less consistent prognostic stratification across cohorts (**Figures 4I–L**). GSTM2 expression was significantly downregulated in patients who experienced BCR compared to those who did not, across both cohorts (*p* < 0.05) (**Figures 4M, N**).

**Figure 4:**
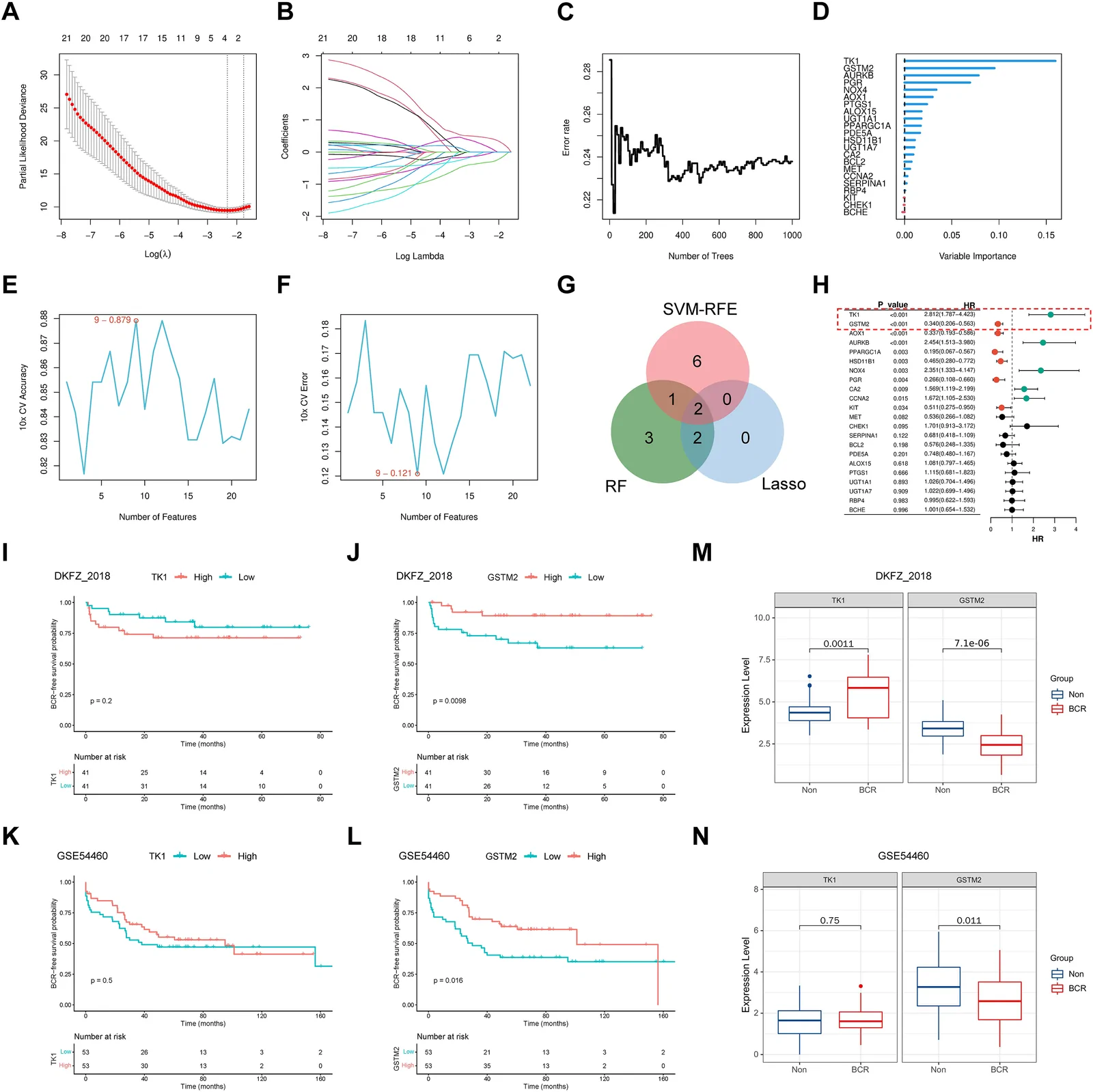
Prognostic gene for PCa using integrated machine learning algorithms. **(A)** Cross-validation curve for the optimal lambda (λ) selection. **(B)** Trace plot of LASSO regression coefficients. **(C)** Error rate by tree count. **(D)** Variable importance ranking. **(E, F)** CV accuracy over feature count **(G)** Overlap among different algorithms. **(H)** Univariate Cox Forest plot. **(I–L)** KM survival curves for BCR-free survival in DKFZ_2018 dataset **(I, J)** and GSE54460 dataset **(K, L)**. **(M, N)** Boxplots comparing the expression levels of TK1 and GSTM2 between Non-BCR and BCR groups in the DKFZ_2018 dataset **(M)** and the GSE54460 dataset **(N)**.

### 3.4 Immune infiltration and drug sensitivity analysis of GSTM2

ESTIMATE analysis served as the initial step to explore GSTM2’s immunomodulatory activity, and TCGA_PRAD samples were stratified by GSTM2 expression. **Figure 5A** shows that GSTM2-high expression correlates with significantly higher ESTIMATE, Stromal, and Immune scores, implying an immunostimulatory role in PCa microenvironment. We next investigated the immune cell infiltration landscape using ssGSEA. A consistent pattern emerged across all three cohorts: GSTM2-high tumors showed significantly increased infiltration of innate immune cells (mast, neutrophils, NK, γδT) and Th1 cells, alongside reduced Tregs (**Figure 5B**). The TCGA_PRAD cohort exhibited broad elevation of both innate and adaptive subsets (including CD8^+^T, B, DCs, and Tem), with downregulation of Th2 and Th17 populations. These findings collectively suggest that GSTM2-high tumors are characterized by an immune-active microenvironment with a favorable antitumor immune orientation. To further evaluate the potential clinical utility of GSTM2, drug sensitivity analysis was conducted based GDSC2 database. Across all three cohorts (TCGA_PRAD, DKFZ_2018, and GSE54460), GSTM2 expression was consistently positively correlated with sensitivity to Bicalutamide, a first-line anti-androgen therapy for PCa (*p* < 0.05) (**Figure 5C**). It indicates that GSTM2 was closely associated with an immunologically active TME and a favorable environment for drug treatment.

**Figure 5:**
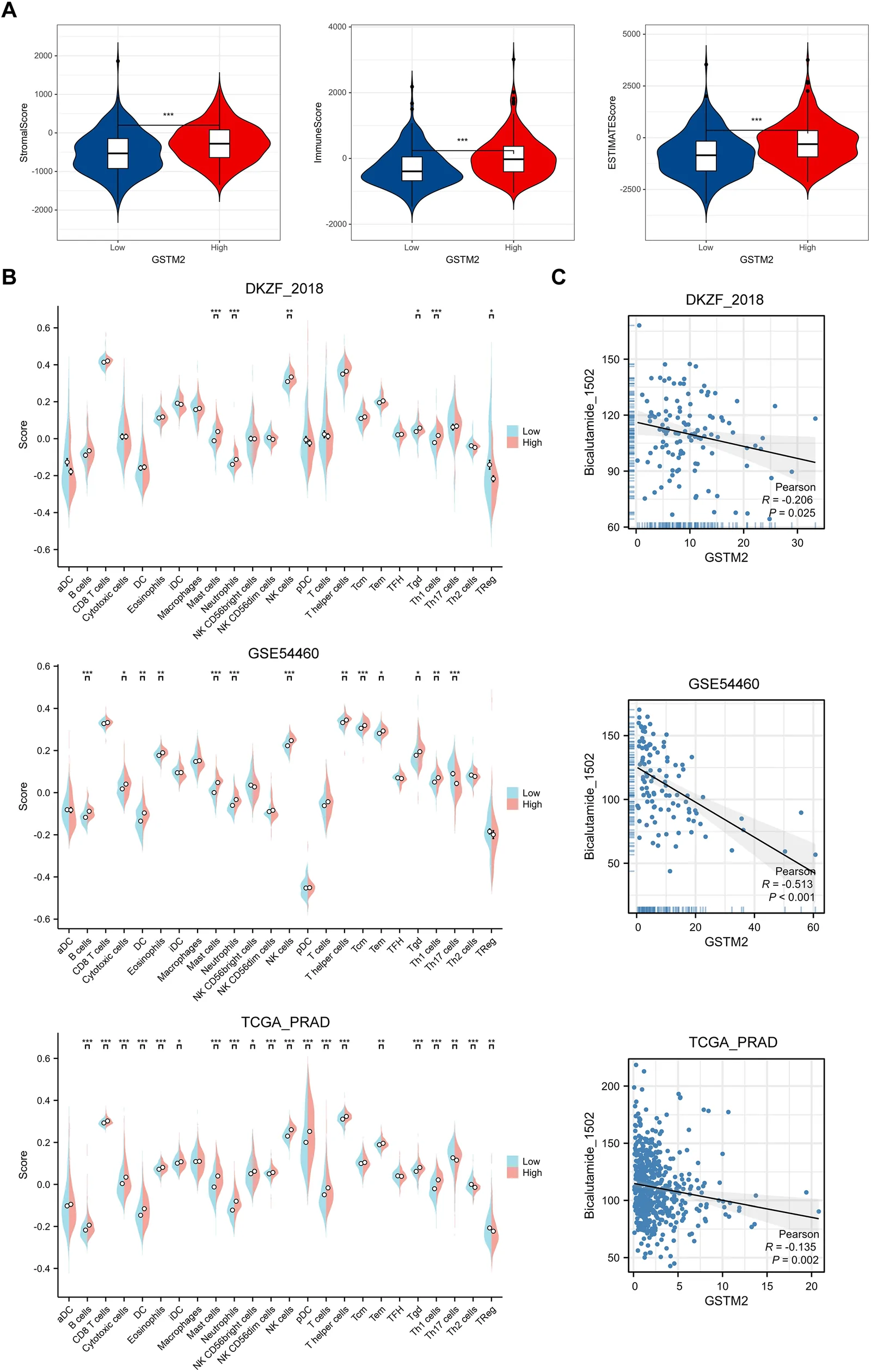
Immune infiltration characterization and drug sensitivity. **(A)** ESTIMATE scores between different groups in TCGA_PRAD. **(B)** ssGSEA results across three cohorts (DKFZ_2018, GSE54460, TCGA_PRAD). **(C)** Correlation of Bicalutamide drug sensitivity with GSTM2 expression across the three cohorts. *p < 0.05, **p < 0.01, ***p < 0.001.

### 3.5 GSTM2 upregulation reduces ROS accumulation

Following dimension-reduction clustering (**Figures 6A, B**), seven major cell types were identified, including T, epithelial (malignant), endothelial, smooth muscle, monocytes, mast, and B cells (**Figures 6C, D**). GSTM2 expression was predominantly restricted to epithelial (malignant), endothelial, and smooth muscle cells (**Figure 6E**). To elucidate GSTM2 function, we stratified epithelial (malignant) cells into GSTM2-positive and GSTM2-negative subsets and identified 2,698 DEGs (**Supplementary Table S10**). Subsequent GSEA revealed that GSTM2-positive cells exhibited significant upregulation of glutathione metabolism and xenobiotic detoxification pathways, indicating enhanced antioxidant capacity, while the respiratory burst pathway, a primary source of ROS production, was markedly downregulated. Pathways related to redox homeostasis and oxidoreductase activity were significantly downregulated, reflecting a low oxidative stress state (**Figure 6F**). All the evidence support that GSTM2-positive tumor cells maintain a quiescent oxidative state.

**Figure 6:**
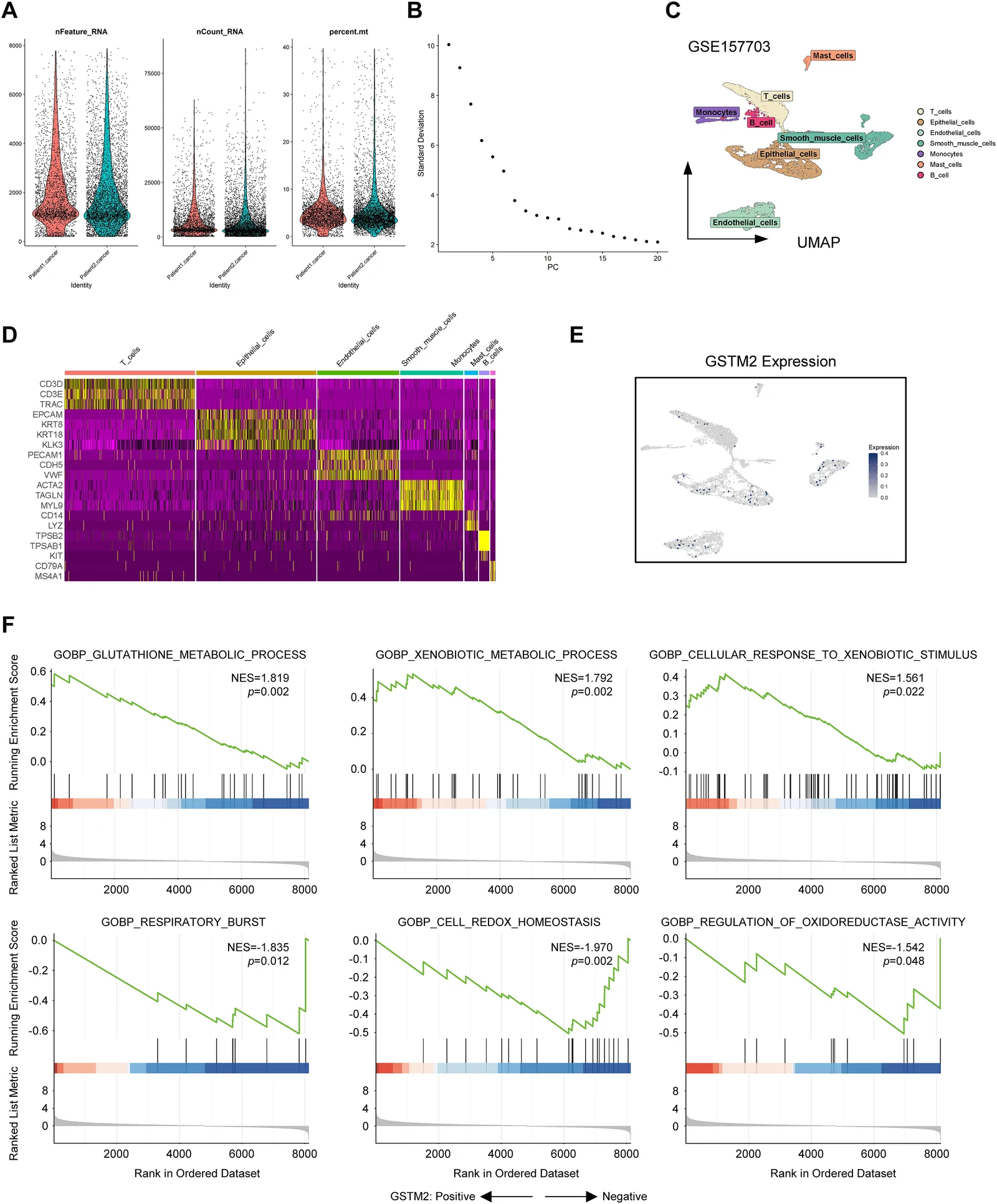
Single-cell profiling reveals GSTM2 in regulating oxidative stress in the TME. **(A, B)** UMAP clustering identifying seven cell types in GSE157703. **(C, D)** Annotation of cell subpopulations. **(E)** GSTM2 expression distribution across cell types. **(F)** GSEA of DEGs between GSTM2-positive and -negative epithelial cells.

### 3.6 Computational analysis of formononetin binding to GSTM2

A binding free energy of −7.5 kcal/mol was observed for the Formononetin–GSTM2 interaction in docking. Interaction analysis revealed that the binding is primarily stabilized by hydrophobic contacts, involving residues Leu122, Leu113, Gln109, Met133, Ala129, Glu125, and Tyr126, while no conventional hydrogen bonds are formed (**Figure 7**). To evaluate the stability of the Formononetin–target complex, we calculated multiple structural metrics from the 100 ns MD trajectory. The RMSD trajectories of the protein and ligand leveled off at 60 ns, with mean values of approximately 0.2 nm and 0.17 nm, respectively, while the ligand alone stabilized after 95 ns at ~0.13 nm (**Figure 8A**). The Rg profiles showed limited variation, suggesting that the overall structural integrity was preserved upon ligand binding (**Figure 8B**). SASA analysis revealed slight variations, suggesting moderate changes in the solvation microenvironment upon binding (**Figure 8C**). Most residues displayed RMSF values below 0.23 nm, reflecting limited local flexibility and a relatively rigid binding pocket (**Figure 8D**). The free energy landscape identified a low-energy basin at PC1 ≈ 0.11 nm and PC2 ≈ 2.2 nm, representing the most stable conformational state of the complex (**Figure 8E**).

**Figure 7:**
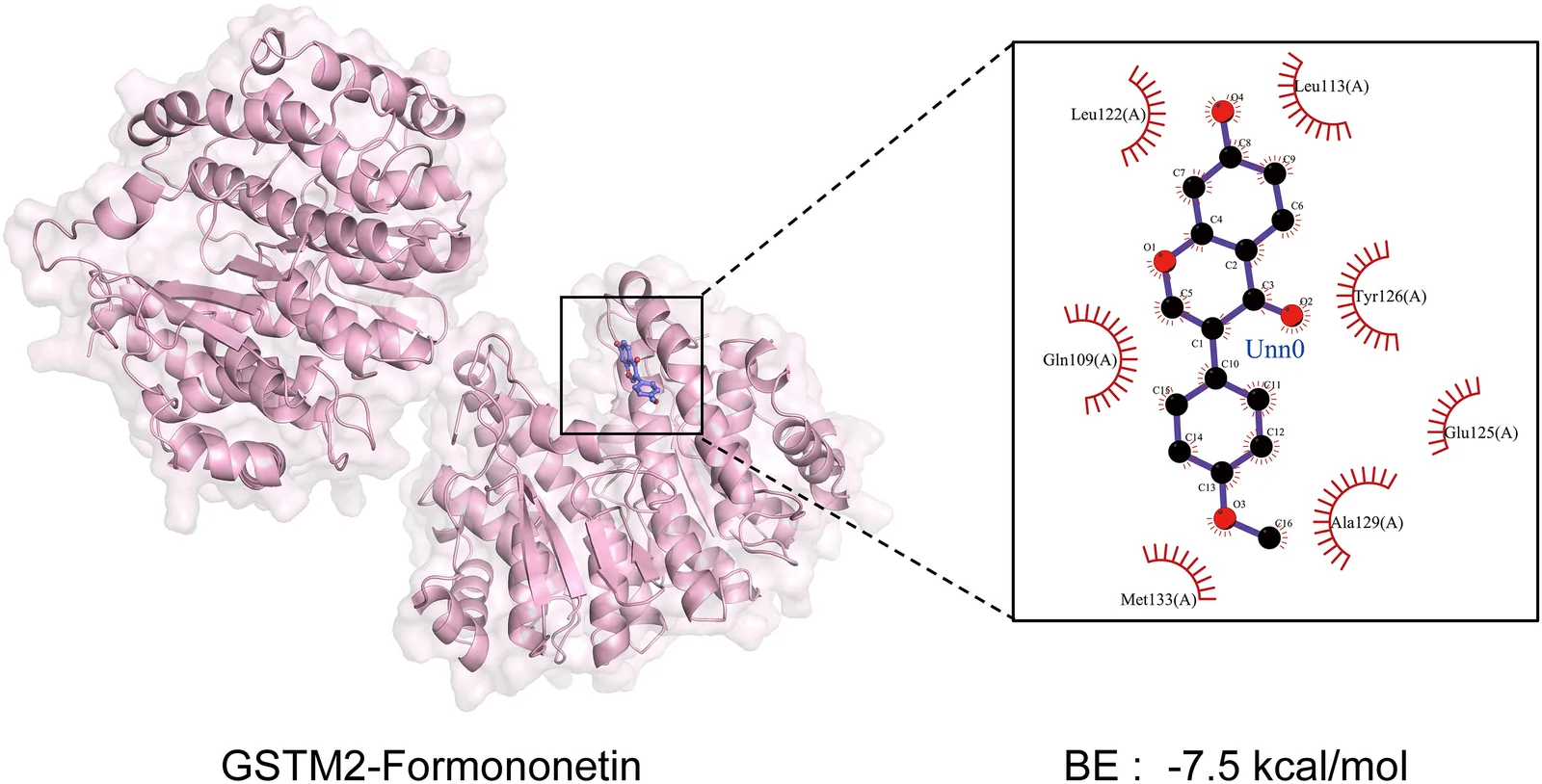
Molecular docking of Formononetin with GSTM2.

**Figure 8:**
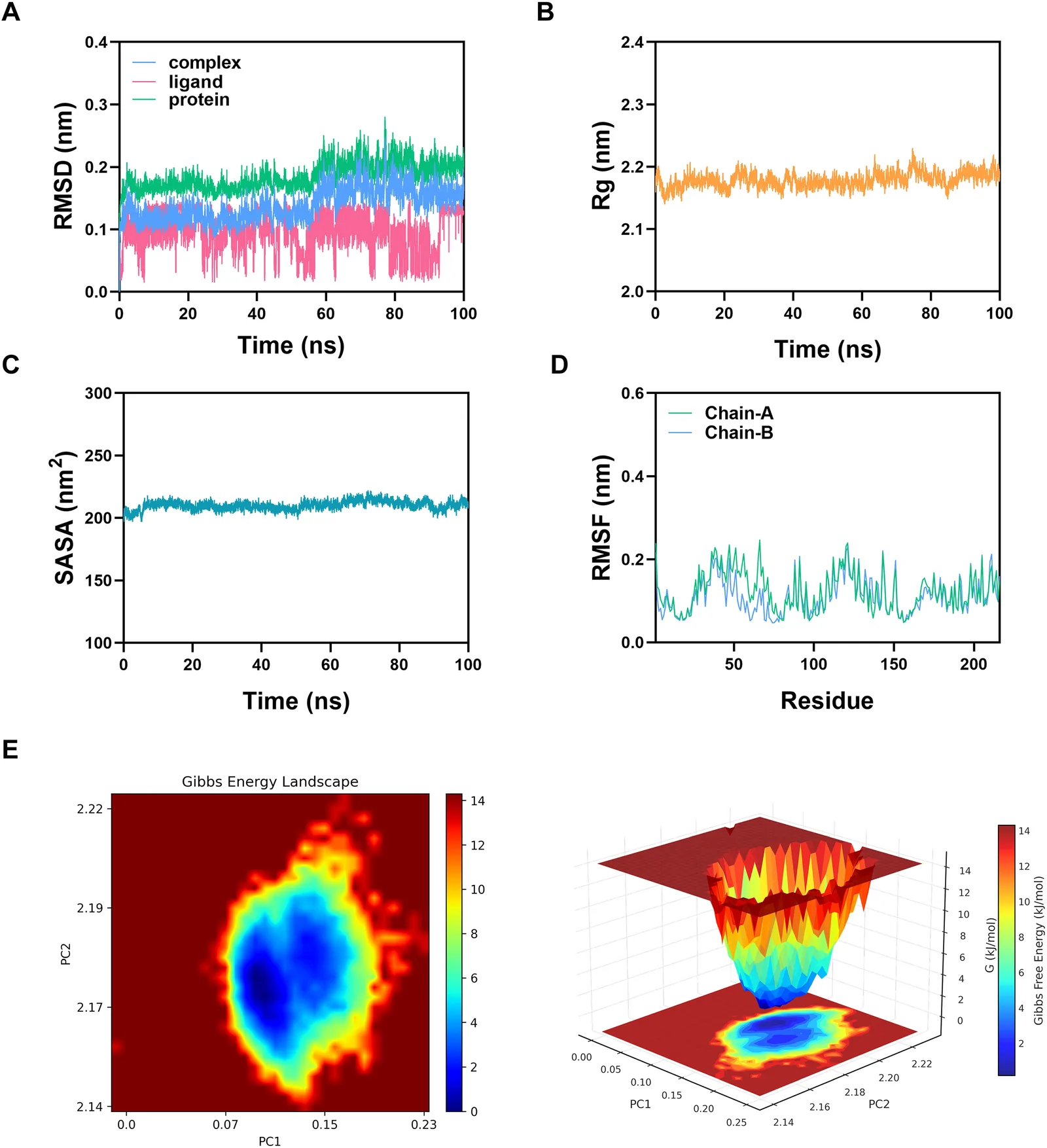
MD of Formononetin-GSTM2. **(A)** RMSD. **(B)** Rg. **(C)** SASA. **(D)** RMSF. **(E)** FEL.

### 3.7 Formononetin inhibits PCa cell malignancy in a GSTM2-required manner

To investigate the effect of Formononetin on GSTM2 expression, we treated LNCaP and VCaP cells with increasing concentrations of Formononetin. This resulted in a dose-dependent upregulation of GSTM2 protein levels (**Figure 9A**), suggesting that Formononetin may enhance GSTM2 stability, possibly through direct binding. We further validated two independent GSTM2-targeting siRNA sequences and used for subsequent functional assays (**Figure 9B**). In CCK-8 and colony formation assays, Formononetin markedly suppressed the proliferation of both LNCaP and VCaP cells, and this inhibitory effect was significantly reversed upon GSTM2 knockdown (**Figures 9C, D**). Consistently, Transwell assays revealed that Formononetin also impairs PCa cell migration and invasion (**Figures 9E, F**).

**Figure 9:**
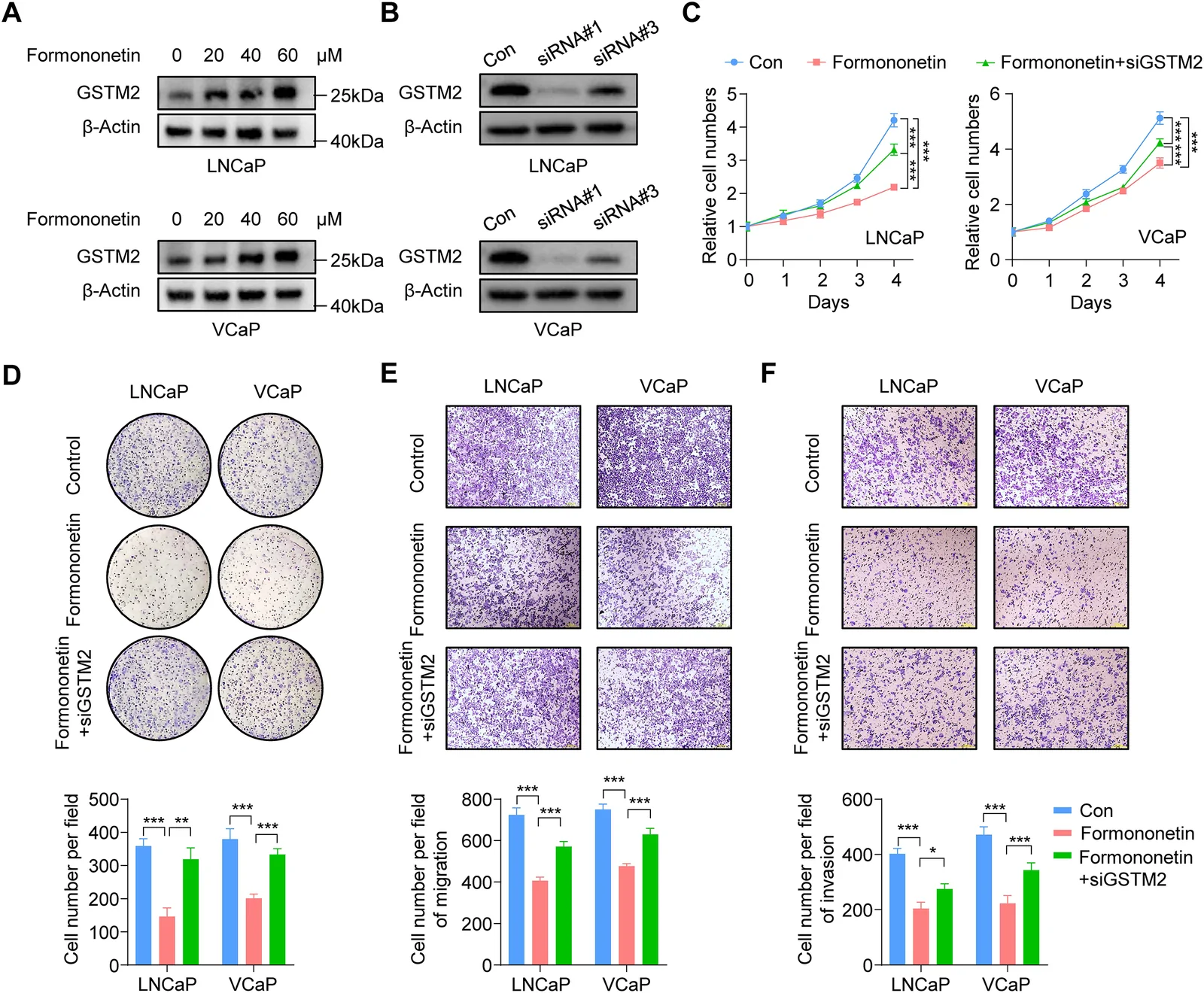
Formononetin inhibits PCa malignancy in a GSTM2-mediated way. **(A)** Formononetin upregulates GSTM2 in dose-dependent. **(B)** Validation of GSTM2 knockdown efficiency. **(C)** CCK-8. **(D)** Colony. **(E)** Migration. **(F)** Invasion. *p < 0.05, **p < 0.01, ***p < 0.001.

### 3.8 GSTM2 mediates formononetin-induced ROS/STAT3-driven chemokine reprogramming in PCa

To model the moderately elevated oxidative stress characteristic of early-stage tumor cells and their microenvironment, we treated LNCaP and VCaP cells with low-dose H_2_O_2_. Under this condition, Formononetin significantly curtailed the H_2_O_2_-evoked ROS response, whereas GSTM2 knockdown significantly reversed this effect, as evidenced by a re-elevation of ROS (**Figure 10A**). Given that ROS act as key signaling molecules, we next examined whether GSTM2-mediated ROS scavenging affects the JAK/STAT axis. As shown in **Figure 10B**, H_2_O_2_ treatment markedly elevated pSTAT3(Tyr705) without altering total STAT1, STAT3 or pSTAT1(Tyr701), indicating selective STAT3 activation under oxidative stress. Formononetin substantially reduced pSTAT3 levels, and this suppression was significantly reversed upon GSTM2 knockdown, demonstrating that Formononetin inhibits STAT3 phosphorylation in a GSTM2-dependent manner. We next assessed CXCL10 and CCL22, two STAT3-regulated chemokines mediating Th1/NK versus Treg recruitment, consistent with our bioinformatics. qPCR and ELISA confirmed that Formononetin regulated CXCL10 and CCL22 expression at transcriptional and secretory levels via GSTM2 (**Figures 10C, D**), revealing a chemokine switch favoring Th1/NK over Treg recruitment.

**Figure 10:**
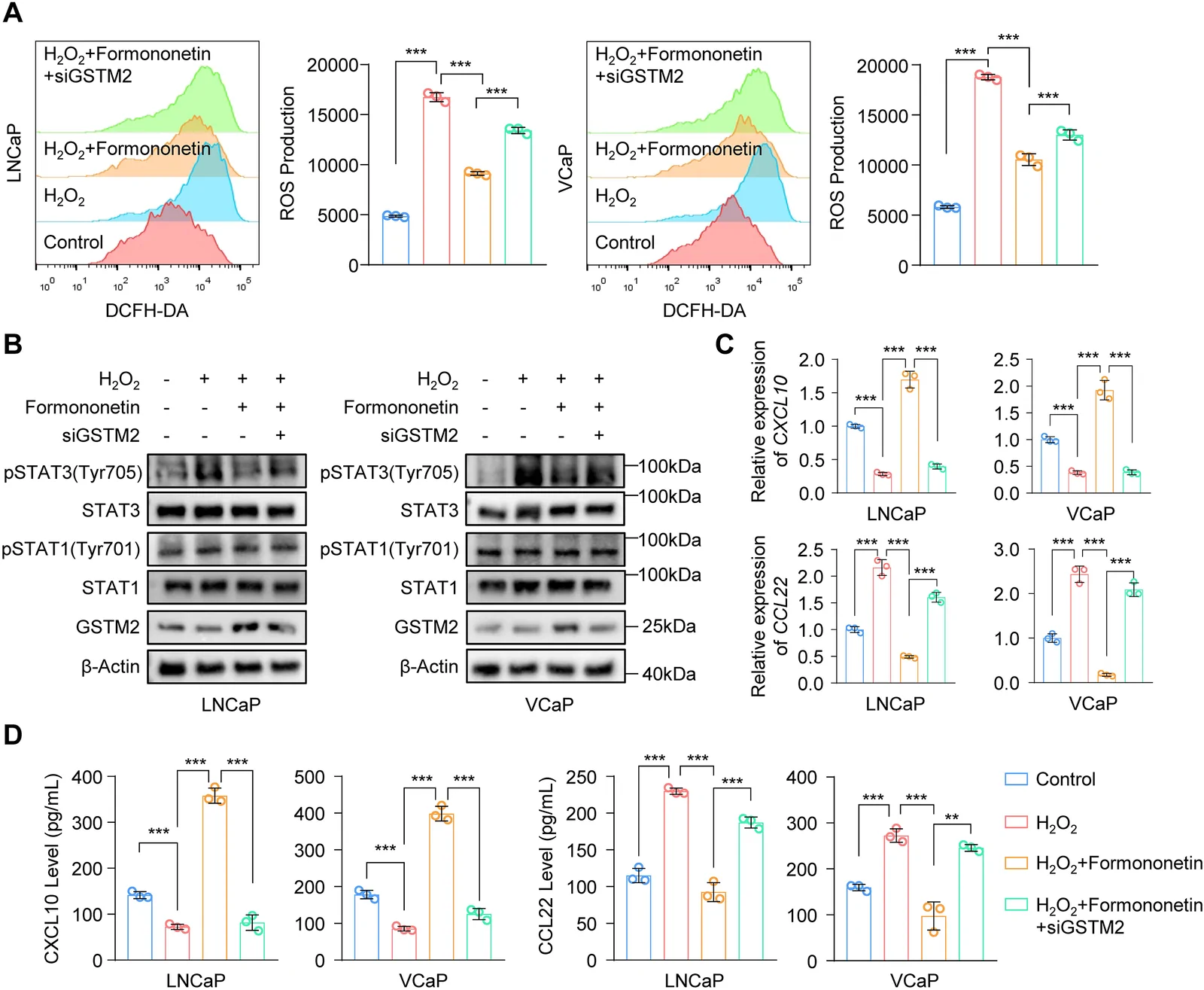
GSTM2 mediates Formononetin-induced ROS/STAT3-driven chemokine reprogramming. **(A)** Intracellular ROS levels in cells under H_2_O_2_, Formononetin, and/or GSTM2 knockdown treatments. **(B)** Western blot analysis of STAT1, STAT3, pSTAT1(Tyr701) and pSTAT3(Tyr705) levels. **(C, D)** qPCR **(C)** and ELISA **(D)** analyses of STAT3-regulated chemokines (CXCL10 and CCL22) at transcriptional and secretory levels. **p < 0.01, ***p < 0.001.

## 4 Discussion

Despite advances in PCa management, BCR remains a major clinical challenge, with few effective strategies available to prevent or delay disease progression. TCM active ingredients, known for their multi-target actions and a well-tolerated nature, are gaining recognition as candidates for cancer prevention and early intervention. In this study, we leveraged a multi-tiered framework to systematically identify GSTM2 as the central target through which Formononetin modulates PCa progression. Compared to another single-cell analysis on the targets of Formononetin, we progressively narrowed down and focus on a verifiable target (46). Among several machine learning algorithms not involved in that study, we specially paid attention to BCR and treated it as a key element. We did not limit into the changes of GSTM2 expression after Formononetin intervention. By combining the GSEA results pointed to ROS in single-cell analysis and different situations of infiltrating cells, we then explored the functional relevance of Formononetin and this target through *in vitro* experiments, coming up with a possible hypothesis.

Formononetin elicits anti-tumor effects through two complementary strategies: direct tumor cell killing and immune microenvironment remodeling. In hepatocellular, breast, and lung cancer models, Formononetin engages PI3K/AKT, EGFR, or mTORC1/SCD1 to suppress proliferation or trigger ferroptosis (7, 47, 48). Studies in other cancers highlight a second mode of remodeling the immune milieu. In osteosarcoma, Formononetin acts through two interconnected mechanisms: it drives ferroptosis via MAZ/GPX4 axis blockade and simultaneously reshapes the immune microenvironment by promoting NK infiltration and reducing exhausted CD8^+^ T cells and neutrophils (49). Astragalus-derived nanoparticle-formulated Formononetin can also reprogram tumor-associated macrophages (TAMs), promotes CD8^+^ T activation and mitigates CD8^+^ T exhaustion (50). Additionally, Formononetin restrains breast cancer proliferation via inhibiting PD-L1 and the STING-NF-κB pathway (51).

Formononetin is closely linked to the ROS/STAT3 signaling axis. In multiple myeloma, Formononetin elevates ROS levels by triggering GSH/GSSG imbalance, thereby inhibiting constitutive activation of STAT3 and STAT5. Consistently, it markedly restrains tumor growth in xenograft mouse models with negligible adverse effects (52). This forms an intriguing parallel to the present study. We demonstrate that Formononetin suppresses STAT3 phosphorylation by scavenging ROS via upregulating GSTM2, whereas prior research reports that Formononetin inhibits constitutive STAT3 activation through ROS induction. These apparently contradictory regulatory patterns imply that the modulatory effect of Formononetin on the ROS/STAT3 axis is cell-type dependent. In normal or premalignant cells, Formononetin preserves redox homeostasis by reinforcing antioxidant defenses. By contrast, in tumor cells with disrupted oxidative equilibrium, Formononetin may initiate cell death signaling by promoting ROS accumulation. Such context-dependent regulation represents a hallmark characteristic of multi-targeted natural products.

Cross-validation of methylation and transcriptomic data indicates that epigenetic silencing of *GSTM2* and subsequent reduction in its expression may serve as a critical driver during the early progression of PCa (13). Notably, *GSTM2* exhibits hypermethylation in more than three-quarters of patients with PCa. Such a high frequency is uncommon among tumor-related epigenetic alterations, implying that GSTM2 inactivation represents an early and prevalent molecular event in PCa pathogenesis. The prognostic significance of GSTM2 is not restricted to PCa. Across multiple datasets of breast cancer, non-small cell lung cancer, and colon cancer, low GSTM2 expression correlates with diminished overall survival (53–55). This pan-cancer evidence indicates that GSTM2 inactivation may constitute a prevalent event in diverse epithelial malignancies. Loss of GSTM2 function, acting as a gatekeeper gene, may facilitate the early expansion of tumor cells. With a focus on immune microenvironment, we revealed that the GSTM2-high group exhibits an immune-active microenvironment, featuring enhanced infiltration of M1 macrophages, CD8^+^ T, NK and Th1 cells, alongside reduced abundance of Tregs, Th2 and Th17 cells. Consistent with the documented immunomodulatory function of GSTM2 in colon cancer (54), these results corroborate our proposed mechanism wherein GSTM2 reshapes immunity through modulation of CXCL10/CCL22.

Intriguingly, Li et al. reported that elevated GSTM2 in enzalutamide-resistant CRPC is associated with anti-androgen resistance (14). This observation seems to conflict with our finding that low GSTM2 expression in early PCa correlates with adverse prognosis, yet it reveals the stage-dependent duality of GSTM2 function. We propose that GSTM2 exerts opposing effects throughout disease progression. In treatment-naive early PCa, impaired GSTM2 gatekeeper function compromises antioxidant defense, enhancing genomic instability and driving malignant transformation. Conversely, under therapeutic stress in CRPC, tumor cells reactivate GSTM2 to counteract treatment-related oxidative stress and acquire drug resistance. This stage-specific functional switch is well recognized in tumor biology; TGF β serves as a classic example, inhibiting proliferation in early lesions while promoting metastasis at advanced stages (56). From an evolutionary viewpoint, tumor cells employ an adaptive strategy regulating GSTM2. Without therapeutic pressure, methylation-mediated GSTM2 silencing accelerates genomic instability and clonal evolution. Upon treatment exposure, GSTM2 re-expression enables tumor cells to survive oxidative damage.

We must acknowledge several limitations. Primarily, the three-group design shows that GSTM2 is necessary for Formononetin’s effect, but we could not rule out the independent phenotypic effect of GSTM2 itself without a siRNA-only group. Second, evidence is lacking *in vivo* to establish the function of GSTM2 in prostate tumorigenesis, along with the therapeutic efficacy of Formononetin, which limits the assessment of GSTM2 as an early preventive target. Third, the immune microenvironment analyses were insufficient evidence, the observed CXCL10↑/CCL22↓ switch is expected to favor the recruitment of anti-tumor immune effector cells over immunosuppressive Tregs. However, more validation should be required to confirm actual microenvironment remodeling, such as Transwell-based immune cell migration assays. The clinical data were derived from public databases, which inevitably introduce patient heterogeneity and selection bias. Future *in vivo* work will employ immunocompetent mouse models of PCa to validate the Formononetin–GSTM2–ROS/STAT3/chemokine axis and to evaluate the translational value of GSTM2 as an early intervention biomarker. In addition, longitudinal cohort studies across different disease stages are needed to verify the stage-dependent functions of GSTM2 and further elucidate its dynamic role in PCa progression. Our data not only position GSTM2 as a downstream target of Formononetin but also delineate a novel mechanism by which the GSTM2–ROS–STAT3–chemokine axis suppresses early PCa progression. This work provides a rationale for targeting GSTM2 in early post-surgical intervention, a strategy that warrants further *in vivo* investigation.

## 5 Conclusion

We identified GSTM2 as a direct target of formononetin in PCa. GSTM2 links Formononetin-mediated redox modulation to CXCL10/CCL22 reprogramming. This remodeling of chemokine signaling helps create an active immune microenvironment.

## Data Availability

The original contributions presented in the study are included in the article/**Supplementary Material**, further inquiries can be directed to the corresponding authors.
